# Enhancement of LacI binding *in vivo*

**DOI:** 10.1093/nar/gkz698

**Published:** 2019-08-09

**Authors:** Manyu Du, Seth Kodner, Lu Bai

**Affiliations:** 1 Department of Biochemistry and Molecular Biology, The Pennsylvania State University, University Park, PA 16802, USA; 2 Center for Eukaryotic Gene Regulation, The Pennsylvania State University, University Park, PA 16802, USA; 3 Department of Chemical Engineering, The Pennsylvania State University, University Park, PA 16802, USA; 4 Department of Physics, The Pennsylvania State University, University Park, PA 16802, USA

## Abstract

Transcription factors (TFs) bind to specific sequences in DNA to regulate transcription. Despite extensive measurements of TFs’ dissociation constant (*K*_d_) *in vitro*, their apparent *K*_d_*in vivo* are usually unknown. LacI, a bacterial TF, is often used to artificially recruit proteins onto eukaryotic genomes. As LacI binds tightly to its recognition site (LacO) *in vitro* with a *K*_d_ about 10 picomolar (pM), it is often assumed that LacI also has high affinity to LacO *in vivo*. In this work, we measured LacI binding in living yeast cells using a fluorescent repressor operator system and found an apparent *K*_d_ of ∼0.6 μM, four orders of magnitude higher than that *in vitro*. By genetically altering (i) GFP-LacI structure, (ii) GFP-LacI stability, (iii) chromosome accessibility and (iv) LacO sequence, we reduced the apparent *K*_d_ to <10 nM. It turns out that the GFP tagging location and the fusion protein stability have a large effect on LacI binding, but surprisingly, chromosome accessibility only plays a mild role. These findings contribute to our quantitative understanding of the features that affect the apparent *K*_d_ of TF in cells. They also provide guidance for future design of more specific chromosomal recruitment through high-affinity TFs.

## INTRODUCTION

Transcription initiation starts with the binding of transcription factors (TFs) to their target sites in promoters and enhancers. The stability and specificity of TF binding are related to their binding affinities, which can be modulated to achieve desirable transcription activities (1–3). TF binding affinity is also an important consideration for synthetic systems where a protein of interest is fused with an exogenous TF to be recruited to its recognition motifs engineered into the genome. In these cases, TFs with high binding affinities are often used to ensure strong recruitment to the target sites while minimizing the off-target effect.

TF binding affinities, or the dissociation constants, *K*_d_, have been extensively measured *in vitro* with purified TFs and DNA containing their recognition sequences (4). *In vivo*, the apparent *K*_d_ (defined as the concentration of a TF at which 50% of the binding sites are occupied), may be further affected by chromatin accessibility, protein stability, interference from other factors and non-specific DNA, etc. The apparent *K*_d_ of most TFs in the cells are unknown, as the *in vivo* measurement is technically challenging. A recent study showed that the apparent *K*_d_ of glucocorticoid receptor (GR) is three orders of magnitude higher *in vivo* than *in vitro* (5), illustrating that *K*_d_ measured *in vitro* may not correctly predict the binding behavior of TFs in cells.

The dimeric *Escherichia coli* lac repressor (LacI) used in this study binds to its recognition site (LacO) with a *K*_d_ of ∼10 pM *in vitro* (6). Because of such high affinity, LacI is widely used in eukaryotic cells for targeted recruitment. The fluorescent repressor operator system (FROS), for example, uses the GFP-LacI fusion to label a chromosome locus containing an array of LacO repeats, and such labeled locus can be tracked as a diffraction-limited fluorescent dot (7,8). The apparent *K*_d_ of GFP-LacI to LacO is an important parameter in this system because it determines the signal-to-noise ratio, i.e. lower apparent *K*_d_ means stronger dot signal with lower background. However, to our knowledge, the apparent *K*_d_ of GFP-LacI has not been measured in eukaryotic cells.

Here, we used FROS to measure the apparent *K*_d_ of LacI binding by varying the concentration of GFP-LacI and quantifying the fraction of bound LacO through imaging. The LacI in the initial system we used shows an apparent *K*_d_ of ∼0.6 μM, four orders of magnitude higher than the *in vitro* value. To reduce the apparent *K*_d_, we genetically altered (i) GFP-LacI structure, (ii) GFP-LacI stability, (iii) chromosome accessibility and (iv) LacO sequence, and evaluated how each of these factors affect LacI binding *in vivo*. By combining the genetic traits that favor binding, we reduced the apparent *K*_d_ of LacI by 40-fold to <10 nM. These results contribute to our understanding of factors that can influence the TF binding *in vivo*. It turns out that the location of GFP tagging and the stability of the fusion protein have a large effect on LacI binding. To our knowledge, this is the first time that the relation between the degradation rate and apparent *K*_d_ is measured experimentally. In contrast, the presence of nucleosome only mildly reduces LacI binding, which is surprising given that nucleosome can change the *K*_d_ of bacterial TFs by ∼5 orders of magnitude *in vitro* (9). Practically, we also provided an improved recruitment system with increased specificity. More specifically, our new FROS setup will allow the use of shorter LacO arrays (less perturbation to the genome) and/or shorter exposure to excitation during fluorescent imaging (less photo damage to the cells) without compromising the signal-to-noise ratio.

## MATERIALS AND METHODS

### Plasmid and strain construction

Standard methods were used to construct the strains and plasmids. Plasmids used in the study were derived from pRS yeast shuttle vectors. Plasmid pSR13 which contains the 256× LacO repeats was provided by Dr Gasser. The LacO templates used in Chromatin immunoprecipitation (ChIP) assays were synthesized by Integrated DNA Technology. Plasmids containing the new 64× or 32× LacO array was cloned through a method developed by Dr Belmont (10). More specifically, we started with a plasmid containing two LacO sites cloned into pRS403 in between SalI and XhoI. We then prepared two digestion reactions on this plasmid: one with BamHI and SalI; the other with BamHI and XhoI. Due to the compatible sticky ends generated by SalI and XhoI, they generated a ligation product that could not be recut with either enzyme. This way, we doubled the number of LacO sites by repeating the digestion and ligation procedures. With increasing copy number of LacO sites, the plasmids became increasingly unstable due to recombination. For the arrays in Figures 4F and 6, the longest we could obtain contain 64× LacO and 32× Osys sites. These arrays were integrated into yeast through a method developed by Rohner *et al.* (11). Yeast strains used in the study were derived from W303. The unstable GFP-LacI was generated by fusing with a *CLN2* PEST degron, which leads to rapid degradation through the SCF^Grr1^–mediated ubiquitination (12). See Supplementary Table SI and II for detailed information of strains and plasmids.

### Fluorescence microscopy

We used the instrumentation and data acquisition platform as described in a previous study (13). Briefly, cells were grown in synthetic complete (SCD) liquid media at 30°C to an OD_660_ ∼0.2, washed, diluted to an OD_660_ ∼0.1, and then transferred onto a coverslip with a SCD agarose pad. After further growth on the pad for 2–3 h, the sample was put under the fluorescent microscope for imaging. Nine z-stacks were taken with an exposure time of 0.2 s and light intensity of 30% at the excitation wavelength of 488 nm. For all our experiments, unlabeled cells were imaged side-by-side with labeled sample to quantify the level of auto-fluorescence.

### Image analysis

We developed Matlab programs to analyze the z-stack fluorescent images (see Supplementary Figure S1 for example) (14). After using the phase image to annotate the cell boundaries, we first generated a composite image where each pixel within the cell boundaries takes on the maximum intensity of this pixel among the z-stack. We analyzed this image to find the location of the chromosome dots. We then went back to the initial z-stack images, generated their intensity scan (minus the auto-fluorescence level of unlabeled cells) and quantified the dot/background intensity at each z position (Supplementary Figure S1). There was a small fraction of cells with extra-bright dots (intensities above 1.8-fold of the average level), presumably due to co-localization of the two LacO arrays after DNA replication, and these dots were discarded in the following analysis.

We next used the highest dot intensity among the z-stacks and the corresponding background intensity to calculate the free protein concentration. For cells with saturated LacI-GFP binding,

$$\begin{eqnarray*}&&\frac{\rm{Total\,intensity\,of\,the\,dot\,at\,saturatinglevel}}{\rm{Total\,backgroundintensity}} \nonumber\\ &&\quad = \frac{{N_{\rm bound}}}{{N_{\rm free}}}=\frac{{N_{\rm bound}}}{\rm{Concentration}\times{\rm area}\times{\rm DOF}}\end{eqnarray*}$$

Here, *N*_bound_ is the number of GFP molecules associated with the LacO array under saturation, which we assumed to be twice the amount of LacO sites (e.g. 512 for the 256× array). The dot intensity is the calculated as the volume underneath the peak in the intensity scan (Supplementary Figure S1B), representing all the photons generated by the bound GFP. For the free LacI-GFP, its concentration is not completely homogenous in the cell due to organelles like vacuole. To best represent the free proteins in the nucleus, we quantified the ‘total background intensity’ as the integrated GFP intensity in a ring enclosing the dot (Supplementary Figure S1C). ‘Area’ in the formula above is the area of the ring; DOF is the ‘depth of field’, which we estimated to be 1.6 μm (Supplementary Figure S2). For cells with unsaturated LacI-GFP binding, the *N*_bound_ and *N*_free_ can simply be computed by comparing the dot/background intensity with the saturated case.

### Validation of our measurement of concentration and apparent *K*_d_

To show our apparent *K*_d_ quantification is internally consistent, we measured the saturated dot intensity on five LacO arrays with different repeat number (32, 72, 144, 256 and 288) as well as the apparent *K*_d_ on two of these arrays (72 and 144). To compare our estimated GFP-LacI concentration to previously published concentration of endogenous proteins, we collected eight datasets of protein copy number per cell measured in synthetic media (15–22), and converted them to concentrations using the average haploid yeast volume 42 μm^3^ (23). Five of these databases were collected using fluorescence measurement of GFP labeled endogenous proteins, and the other three used quantitative mass spectrometry. We imaged two GFP-labeled endogenous proteins, Ser2 and Rnr4, and compared their fluorescence intensity and reported concentration to those of GFP-LacI.

### ChIP assay

The ChIP protocol was modified from a previously described method (13). Cells were grown in 50 ml SCD to reach OD660 ∼0.4 and then crosslinked by formaldehyde (final concentration 1%) for 20 min. After quenched with 2.7 ml of 2.5 M glycine, these cells were harvested by centrifugation at 2000 rpm for 10 min at 4°C. The cells were then resuspended in 250 μl of FA-lysis buffer containing proteinase inhibitors and disrupted by 250 μl of glass beads for 30 min. We punched a hole at the bottom of the tube and centrifuge the sample to get rid of the glass beads, and the whole cell extract was sonicated to fragment chromatin to lengths typically in between 200 and 800 bp. We centrifuged the sonicated samples at 14 000 rpm for 20 min to get rid of the cell debris and transferred the supernatant to a new tube, where it was incubated with anti-GFP antibody (Abcam 250) for 10–12 h at 4°C and then with Protein A/G PLUS-Agarose beads for 4 h (Santa Cruz Biotechnology, sc-2003). An aliquot of the supernatant was saved for input control. We extracted DNA from the input and immunoprecipitated samples and quantified them by quantitative polymerase chain reaction (qPCR) analysis. All ChIP experiments were repeated three times.

### Measurement of nucleosome occupancy using DNA methylation

Protocols were modified from a previously described method (24). Strains containing the LacO binding sites and M. cviPI methyltransferase were grown in YPD media overnight to saturation. They were washed twice with water on the next morning and then diluted to an OD_660_ of 0.1 in 40 ml SCD media. After 2–3 h when the OD_660_ was reached ∼0.2, we added 40 ml fresh SCD and split each culture into two. We added 4 l of 1 mM β-estradiol to one culture for induction of the DNA methylation and 4 μl of 100% ethanol to the other culture to serve as the control. After 2 h, we purified the genomic DNA through standard phenol extraction. The genomic DNA was then treated with the reagents in the EZ DNA methylation-lightning kit (ZYMO RESEARCH) so that the unmethylated cytosine is converted into uracil. We then performed PCR on the converted genomic DNA template and sequenced the PCR products of regions of interest through Sanger sequencing. The nucleosome occupancy is indicated by the phred value of thymine. The higher conversion rate from cytosine (C) to thymine (T) represents higher nucleosome occupancy.

## RESULTS

### Large apparent *K*_d_ of LacI in a commonly used FROS

**Figure 1.**
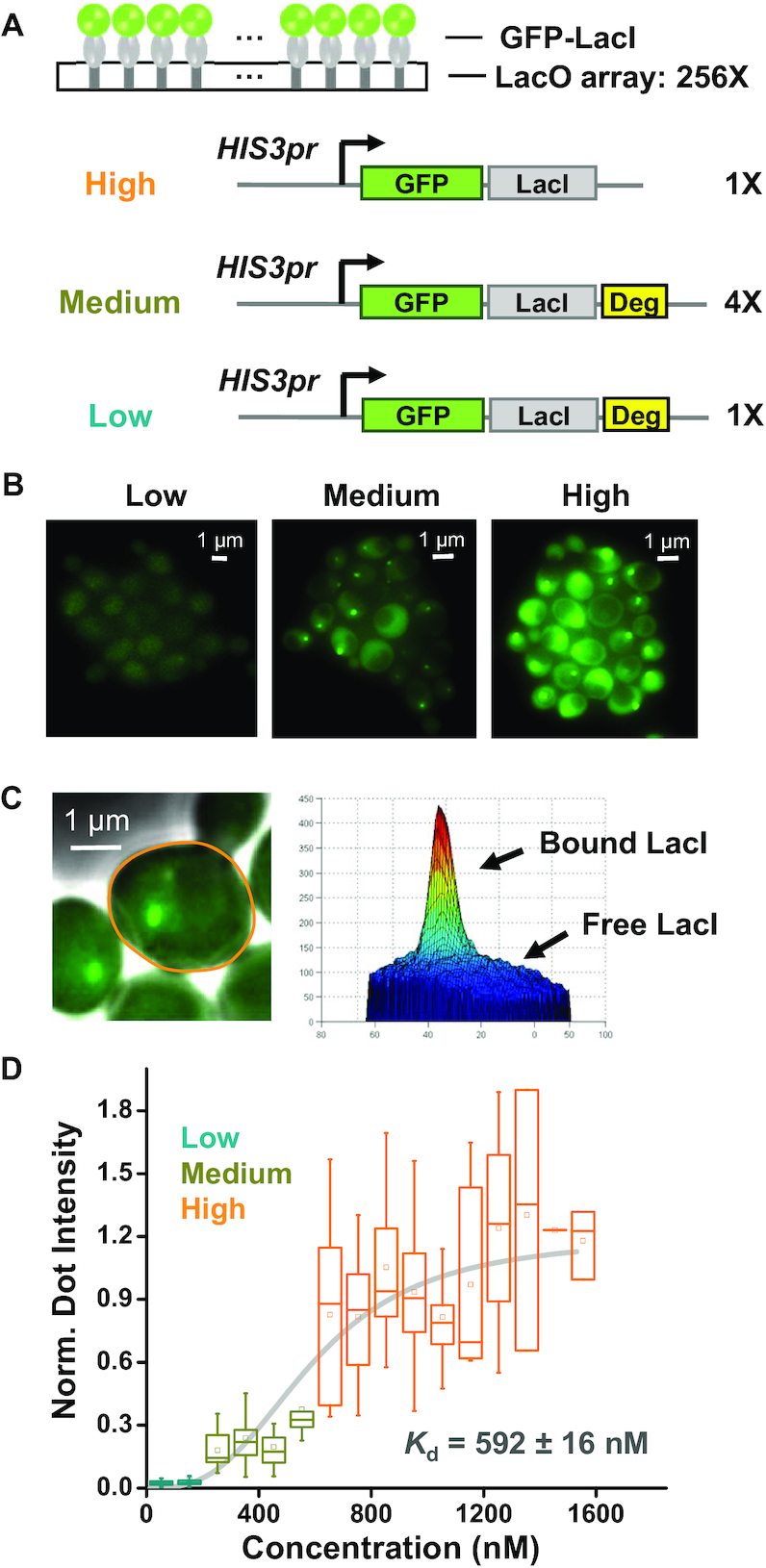
LacI shows large apparent *K*_d_ in a commonly used FROS. (**A**) Scheme of FROS where GFP labeled LacI is recruited to arrays of LacO with 256 repeats (256×; same notation is used below). GFP-LacI is driven by *HIS3* promoter ± the destabilization sequence (deg). These constructs were integrated into the yeast genome with different copy numbers to generate variable concentrations of the fusion protein. (**B**) Fluorescent images of the FROS at different GFP-LacI concentrations. All images are shown with the same brightness and contrast (same as below). (**C**) Measurement of the amount of bound versus free GFP-LacI. The right panel shows the intensity profile of the highlighted cell in the left panel, where the peak reflects the fluorescence generated by the bound GFP-LacI, and the background by the free ones. See Supplementary Figure S1 and ‘Materials and Methods’ section for detailed quantification method. (**D**) Measurement of the dissociation constant, *K*_d_. The box plot shows the normalized dot intensity versus background GFP-LacI concentration measured in individual cells (total number of cells: *N* = 268). The data were binned into 100 nM windows and fitted by a Hill function with *K*_d_ = 592 ±16 nM and hill coefficient *n* = 2.93 ± 0.42. Error bars shown here represent 5th to 95th percentile (same as below).

We defined the ‘apparent *K*_d_’ as the concentration of LacI at which 50% of the LacO sites are occupied. To measure the apparent *K*_d_*in vivo* using FROS, we constructed strains containing a 256× LacO array and GFP-LacI at different concentrations (Figure 1A), The LacI protein used in the FROS lacks the tetramerization domain, and under physiological salt condition, its affinity to the wt LacO site *vitro* has a *K*_d_ of ∼10 pM (6). Using the original construct with GFP-LacI driven by the *HIS3* promoter, the LacO array can be visualized as bright ‘chromosome dots’ with high GFP background (Figure 1B). To lower the steady-state level of GFP-LacI, we generated a destabilized version of GFP-LacI (GFP-LacI-degron) (25,26), which reduces the background GFP intensity by 10-fold (Figure 1A and B). We also constructed a strain with intermediate GFP-LacI concentration by integrating four copies of the GFP-LacI-degron into the genome. Besides these genetic manipulations, we took advantage of the natural cell-to-cell variability in the GFP-LacI concentration for the apparent *K*_d_ measurement.

We quantified the intensity of the chromosome dot (representing the amount of bound GFP-LacI) and the background intensity (representing the amount of free GFP-LacI in the vicinity of the dot) for each single cell (Figure 1C; Supplementary Figures S1 and 2). In yeast cells containing the stable GFP-LacI, the dot intensity is not correlated with the background intensity (Figure 1D), indicating that LacI binding reaches saturation. Assuming the saturation condition represents full occupancy of LacO, there should be 512 bound GFP-LacI (each LacO binds a LacI dimer), we can calculate the copy number of the free GFP-LacI by comparing the background intensities with the saturated dot intensity (‘Materials and Methods’ section). In the low-background strain containing 1× GFP-LacI-degron, the GFP-LacI concentration was estimated to be ∼100 nM. Despite the fact that this concentration is much higher than the reported *K*_d_*in vitro*, we could barely see chromosome dots in this strain (Figure 1B). By fitting the dot intensity vs GFP-LacI concentration with a Hill function, the apparent *K*_d_ turns out to be ∼600 nM, four orders of magnitude higher than the *K*_d_*in vitro* (Figure 1D).

We carried out a number of tests to validate the *K*_d_ measurement above. First, for five LacO arrays with different number of LacO repeats, we expressed high level of GFP-LacI and quantified the saturated dot intensities. As expected, the intensities scale linearly with the LacO repeat number (Pearson correlation: *R* = 0.99) (Supplementary Figure S3A). Second, when we expressed GFP-LacI at different levels and measured the apparent *K*_d_ using two of these arrays, we found that the apparent *K*_d_ is not affected by the array size (Supplementary Figure S3B and C), showing that our measurements are internally consistent. To get an independent assessment of the accuracy of the measured concentration, we imaged the GFP-labeled endogenous Ser2 and Rnr4 proteins together with *HIS3pr-GFP-LacI*, and compared their fluorescence levels and concentrations (‘Materials and Methods’ section). Despite the fact that the published Ser2 and Rnr4 concentrations were measured with methodologies different from ours, the fluorescence intensities from the three strains are largely proportional to the concentration of the three proteins (Supplementary Figure S3D). These data verify our method of quantifying concentration and apparent *K*_d_.

To understand which factors compromised LacI binding *in vivo*, we next genetically modified the structure and the stability of the fusion protein, chromatin accessibility, and the intrinsic binding affinity of LacO to quantitatively examine how they affect the apparent *K*_d_ of LacI.

### The apparent *K*_d_ of LacI is reduced by fusing GFP to the C-terminus of LacI

**Figure 2.**
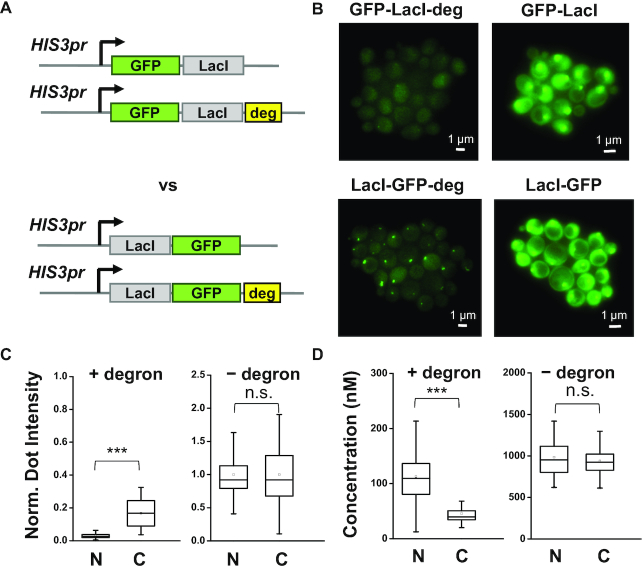
The apparent *K*_d_ of LacI is enhanced by fusing GFP to the C-terminal of LacI. (**A**) Construct of the fusion proteins with GFP linked to either the N- or C-terminal of LacI. (**B**) Fluorescent images of the FROS with different fusion proteins in A. (**C** and **D**) Normalized dot intensity (C) and free protein concentration (D) in the four strains containing GFP fused to the N- or C-terminal of LacI with (left panel) or without (right panel) the degron sequence (*N* = 137, 29, 71 and 48 for the four strains, respectively). The dot intensity increases by 5.3-fold (*P*-value < 0.0001) after moving the GFP to the C terminus, while the background concentration is reduced by 2.5-fold (*P*-value < 0.0001).

The crystal structure of the LacI protein shows that its DNA binding domain is located at the N-terminus (27). In the original construct, the GFP was fused to the N-terminus with a largely unstructured linker region, indicating that GFP in some orientations may sterically block LacI binding (Supplementary Figure S4). We therefore moved the GFP to the C-terminus and imaged the chromosome dots on the same 256× LacO array (Figure 2A). At high concentration, GFP-LacI and LacI-GFP show essentially the same dot intensity, consistent with saturated binding (Figure 2B and C). However, at low concentration, LacI-GFP shows higher binding with lower free protein background (Figure 2C and D). These results clearly show that the binding of LacI is enhanced by labeling GFP at its C-terminus.

### The apparent *K*_d_ of LacI is reduced by using stabilized version of LacI

**Figure 3.**
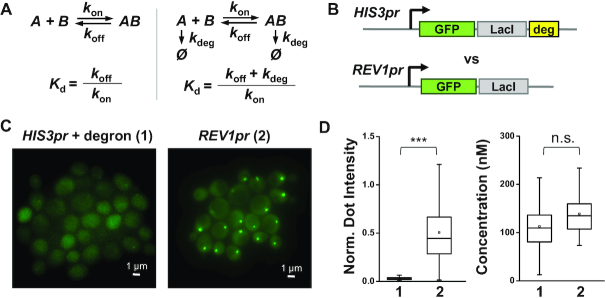
The apparent *K*_d_ of LacI is enhanced by using a stabilized version of LacI. (**A**) Analysis of a simple binding reaction with or without active degradation shows that a high degradation rate can significantly increase the effective *K*_d_. *k*_on_: binding rate, *k*_off_: dissociation rate, *k*_deg_: degradation rate. (**B**) Generating low concentration of stable GFP-LacI using a promoter of low activity (*REV1pr*). (**C**) Fluorescent images of the FROS on the original 256× LacO array with the constructs in B. (**D**) Quantification of the normalized dot intensity (left panel) and the free protein concentration (right panel) in these two strains (*N* = 137 and 57, respectively). The dot intensity increases by 15-fold (*P*-value < 0.0001) with the stable GFP-LacI, while the background concentration is about the same.

Stability of a protein may also affect its binding kinetics. Theoretical analysis of a simple binding reaction with or without active degradation shows that a high degradation rate can increase the apparent *K*_d_ (Figure 3A). Conjugating the *CLN2* degron to a heterologous protein results in a protein half-life of 12 min (*k*_deg_ = 9.6 × 10^−4^s^−1^) (28). At physiological salt concentration, the association rate of dimeric LacI is diffusion-limited (10^7^–10^8^ M^−1^s^−1^) (29,30), and the *k*_off_ should be in the range of 10^−4^ – 10^−3^ s^−1^. Assuming the degradation rate is comparable in the nucleus and in the cytoplasm, these values suggest that the high degradation rate can increase the apparent *K*_d_ by up to 10-fold.

To eliminate active degradation while maintaining low steady-state protein level, we used a weaker constitutive promoter, *REV1* promoter, to drive the expression of the stable GFP-LacI (Figure 3B) (31). In comparison to GFP-LacI-degron driven by the *HIS3* promoter, this construct increases the GFP dot intensity by 15-fold while maintaining similar background level (Figure 3C and D). Such increase in the LacI binding is consistent with our expectation above.

### The apparent *K*_d_ of LacI is mildly reduced by increasing the accessibility of chromosome

**Figure 4.**
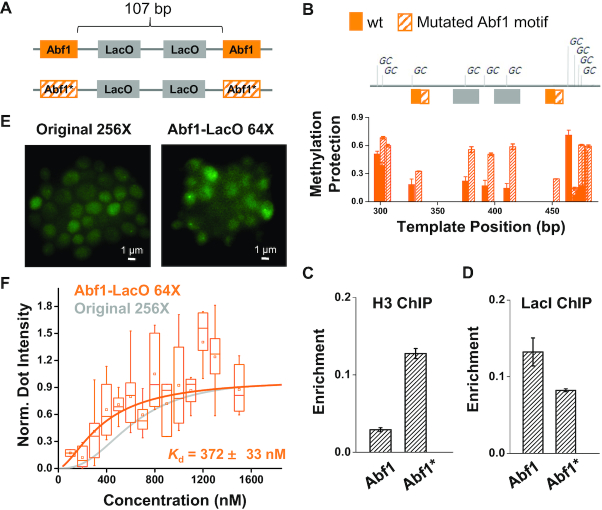
The apparent *K*_d_ of LacI is mildly enhanced by increasing chromatin accessibility. (**A**) LacO templates for testing the nucleosome effect on LacI binding. This template contains two LacO sites flanked by two consensus or mutated (*) Abf1 motifs. (**B**) Methylation protection assay over the two templates in A in strains lacking LacI. For every ‘C’ in the ‘GC’ context, its conversion level to ‘T’ is plotted. Only the unmethylated (protected) ‘C’s will be converted to ‘T’. The conversion level thus corresponds to the protection level against methylation. The three ‘GC’s over the LacO sites are more protected on the template with Abf1* (hatched bar) than with wt Abf1 (solid bar), indicating that it has higher nucleosome coverage. (**C**) ChIP measurement of Histone H3 over the two templates in A. Consistent with B, H3 is more enriched on the template with Abf1*. (**D**) ChIP measurements of LacI over the two templates. LacI shows higher enrichment on the LacO flanked by the wt Abf1 sites. Error bars shown here represent standard error of the mean (SEM) among three biological repeats. (**E**) Imaging data of *HIS3pr*-GFP-LacI-degron binding on Abf1-LacO array (64× LacO) versus original LacO array (256×). Abf1-LacO array is generated by duplicating the template with the wt Abf1 in A with 32 repeats. We observed brighter dots over the Abf1-LacO array despite the reduced number of LacO sites. (**F**) *K*_d_ measurement of the GFP-LacI binding on the two arrays above (*N* = 165 for the Abf1-LacO array, and 268 for the original array). The Abf1-LacO array mildly reduces the apparent *K*_d_ (*P-* value < 0.0001).

Chromosome accessibility can be another crucial factor that affects TF binding. Many TFs have difficulty accessing their binding sites embedded in the nucleosomes (32–34). The *K*_d_ of two TFs, LexA and Gal4, is increased by four to five of magnitude *in vitro* when the same binding motif is covered by a nucleosome (9). We therefore investigated if nucleosomes have the same effect on LacI *in vivo* by perturbing the nucleosome occupancy on LacO and probing its effect on LacI binding. Abf1 is a strong nucleosome-displacing factor in yeast that antagonizes nucleosome formation near its binding site (35–37). On a new LacO template, we added an Abf1 binding motif to each side of two LacO sites (Figure 4A). The distance between the two Abf1 motifs is 107 bp, shorter than the length of a single nucleosome, and therefore, we expect the LacO sites in-between to be nucleosome-free. For comparison, we generated another template containing 2-bp mutation in the Abf1 motifs (Figure 4A).

We measured the nucleosome occupancy on the two templates above in strains lacking the LacI protein. The assay commonly used for nucleosome mapping, micrococcal nuclease digestion followed by stacking PCR (38,39), is not suitable here because of the ambiguity introduced by the repetitive sequences. Instead, we used a methylation assay where nucleosome or other chromosome-bound proteins are detected based on protection against DNA methylation (‘Materials and Methods’ section) (24). LacO sites with the consensus Abf1 motifs are more susceptible to methylation (less protected) than the ones with the mutated Abf1 motifs (Figure 4B). The level of protection in these two cases, 0.2 and 0.6, are consistent with regions known to be exposed or embedded under the nucleosome (Supplementary Figure S5). To confirm the difference in methylation susceptibility is indeed due to nucleosomes, we also conducted histone H3 ChIP over the LacO sites with consensus or mutated Abf1 motifs. Higher histone enrichment was found in the latter case (Figure 4C). Together, these data suggest that we have generated two templates with different nucleosome coverage over the same LacO sites.

We then introduced the LacI protein into these strains and performed ChIP assay to measure the level of LacI binding on the Abf1/Abf1*-LacO templates (‘Materials and Methods’ section). There is a mild but significant improvement of LacI binding on the template containing the consensus Abf1 sites (*P*-value = 0.021) (Figure 4D). To confirm the ChIP results, we amplified the templates into an array with 64× LacO sites and performed imaging to quantify the bound GFP-LacI-degron. Despite the fact that the new template contains four times less LacO sites than the original 256× array, we detected comparable or even brighter chromosome dots with GFP-LacI-degron driven by the *HIS3* promoter (Figure 4E). The dot intensity versus free protein curve yields an apparent *K*_d_ of 372 ± 33 nM, smaller than that on the original LacO template (apparent *K*_d_ = 592 ± 16 nM; *P*-value < 10^−4^) (Figure 4F). We did the same set of experiments on templates with different linker sequences between LacO and Abf1 sites, and reached the same conclusions (Supplementary Figure S6). These results suggest that nucleosomes have a mild inhibitory effect on LacI binding in the cells.

### The apparent *K*_d_ of LacI is reduced by using symmetric LacO sites

**Figure 5.**
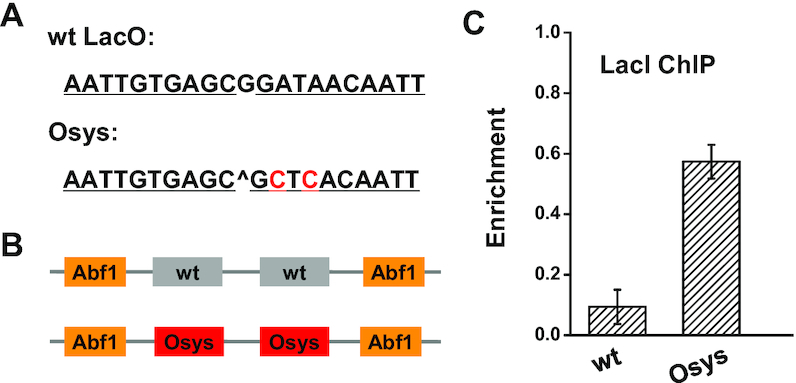
The apparent *K*_d_ of LacI is enhanced by using a symmetric Lac operator sequence. (**A**) DNA sequences of the wildtype Lac operator (wt LacO) and the symmetric Lac operator (Osys). (**B**) Templates used for the LacI binding measurements. The templates contain two wt or symmetric LacO. (**C**) LacI enrichment on the two templates measured by ChIP assay. Osys increases the LacI binding by 6-fold. Error bars shown here represent SEM of three repeats.

Previous studies have identified a symmetric Lac operator (Osys) that binds LacI with higher affinity than the wild-type version *in vitro* (*K*_d_ is reduced by 5-fold) (Figure 5A) (40–42). To examine if LacI also shows stronger binding to Osys *in vivo*, we swapped the two wild-type LacO sites flanked by Abf1 with Osys (Figure 5B) and evaluated LacI binding with ChIP. We observed a 6-fold increase in the LacI enrichment on Osys than the wild-type LacO (Figure 5C), which is comparable to what was measured *in vitro*. We also conducted the same ChIP experiments on templates containing different linker sequences, and LacI consistently binds Osys better than the wild-type LacO (Supplementary Figure S7). We conclude that Osys leads to robust increase in LacI binding *in vivo*.

### A combined system reduced the apparent *K*_d_ of LacI to <10 nM

**Figure 6.**
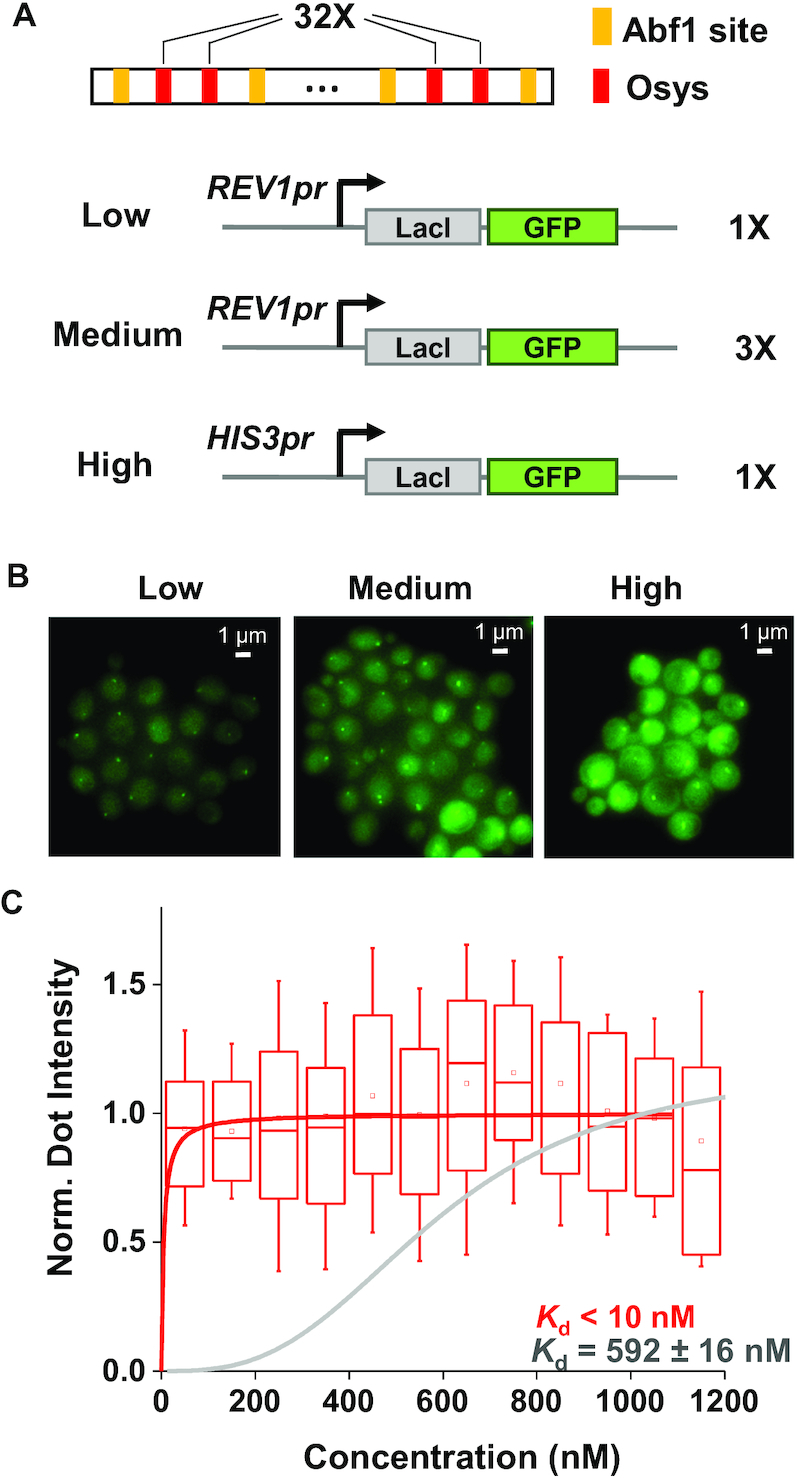
The apparent *K*_d_ of LacI is reduced by over 60-fold after combining all the genetic traits that favor LacI binding. (**A**) Scheme of a new FROS, including the *REV1pr* driving the expression of a stable LacI-GFP and an array of 32 copies of Osys flanked by Abf1 sites. (**B**) Fluorescent images of the new FROS at different GFP-LacI concentrations. (**C**) Apparent *K*_d_ measurement of the new FROS. The normalized dot intensity is plotted against the background GFP-LacI concentration in individual cells (*N* = 112) with a bin size of 100 nM. The LacO array is fully occupied even at the lowest LacI concentration, indicating that the apparent *K*_d_ of the system is <10 nM. A binding curve of *K*_d_ = 5 nM is shown for comparison.

We next combined all the genetic manipulations above to generate a new FROS with much higher occupancy of LacI at low concentration. This system includes stable LacI-GFP driven by the *REV1* promoter, as well as a LacO template containing 32× Osys flanked by Abf1 (Figure 6A). We again generated three strains with different LacI-GFP level (low and medium: 1× and 3× *REV1pr-LacI-GFP*, and high: *HIS3pr-LacI-GFP*) and measured the bound vs free GFP intensity in single cells. LacI shows similar binding in all three strains regardless of the LacI-GFP concentrations (Figure 6B and C). We further reduced the LacI-GFP level by 30% by putting a single copy of *REV1pr-LacI-GFP* into a diploid yeast strain. Even in this condition, the binding of LacI is near-saturated (Supplementary Figure S8A). Given that we did not see a significant drop in the dot intensity with LacI-GFP concentration as low as 30 nM, the apparent *K*_d_ should be <10 nM (Figure 6C and Supplementary Figure S8B), more than 60-fold lower than what we started with in Figure 1. The improved signal-to-noise ratio here allows chromosome dots to be visualized with lower excitation (and thus, lower photo-damage to the cells) and/or less LacO repeats (less perturbation to the genome). The way we improved FROS will provide guidance for future design of more specific chromosomal recruitment through high-affinity TFs.

## DISCUSSION

The apparent *K*_d_ of TFs *in vivo* is a parameter of high functional relevance. The DNA binding domain of a TF is usually conjugated to a functional domain (e.g. activation domain), and how specific such functional domain acts on the target sites vs the rest of the genome is determined by the ratio between the bound and free TFs. TFs with low apparent *K*_d_ can occupy target sites at low concentration, leading to high specificity. The LacO/LacI system is a popular choice for artificial recruitment in eukaryotic species because of its high affinity *in vitro*. However, to our knowledge, the apparent *K*_d_ of LacI *in vivo* has not been carefully evaluated in these species. Based on our results, we suspect that in some studies, the LacI fusion proteins were expressed to high levels that masked the weak binding of LacI, but such solution may also increase the background noise and reduce the sensitivity of the measurement.

In a few past studies, *in vivo* binding of TFs were measured using two types of methods. The first one is an ‘equilibrium’ method, which measures the occupancy of the binding site at different TF concentrations using footprinting assay (43,44). To get robust signal in this assay, a large amount of TF binding sites needs to be introduced into the cells on plasmids. The chromosome configurations on these DNAs may be distinct from the native genome and thus affect the TF binding. Alternatively, it can be measured by the ‘kinetic’ method, where sparsely labeled TFs are tracked in real time to reveal *k*_on_ and *k*_off_ (45–47). This method has been used to measure LacI dynamics in live *E. coli* cells (47–50). Besides being technically challenging, this method usually cannot pinpoint a single genomic locus, and the *K*_d_ measured this way often reflects the binding averaged over different genomic sites. In our study, taking advantage of the FROS setup, we developed a new strategy to measure the apparent *K*_d_ by simultaneously visualizing the proteins that are either bound on specific chromosomal sites or free in the nucleus. This method in principle can be applied to other TFs.

Starting with a commonly used FROS construct, we found that LacI binding to LacO is very weak in the yeast nucleus (apparent *K*_d_ ∼0.6 μM). We need to point out that there are a few factors that may confound this estimation. First, we assumed that LacO sites are 100% occupied by LacI-GFP upon saturation. If only a fraction of LacO is occupied instead, our assumption above would lead to an overestimation of the background LacI-GFP concentration, and therefore an overestimation of apparent *K*_d_. Given that a LacO array as short as 32× can yield bright chromosome dots (Figure 6), the bound fraction is unlikely to be much smaller than 100%. Second, a fraction of GFP molecules may be fluorescently invisible because they are immature or photo-bleached. These proteins should be present in the bound and free population with the same proportion, and therefore will not affect the estimated free protein concentration and *K*_d_. Third, the expression of GFP-LacI may generate free GFPs unlinked to LacI, which will cause an overestimation of *K*_d_. This is an unlikely scenario as LacI is translated efficiently in yeast. Indeed, a number of tests have validated our quantification of LacI concentration and apparent *K*_d_ (Supplementary Figure S3).

We demonstrated that multiple genetic features can be manipulated to decrease the apparent *K*_d_, including fusion protein structure, stability, chromatin accessibility and binding site sequence. Out of these measures, only the last one directly impacts the non-covalent interactions at the protein–DNA interface. The other ones affect the accessibility of the DNA (due to nucleosome coverage) or the protein (due to degradation or tag interference). Some of these effects, e.g. those caused by protein stability and Osys, are consistent with expectations. However, we were surprised to observe that nucleosome only has a mild inhibitory effect on LacI binding. Nucleosomal DNA was shown to be highly inaccessible for most TFs except the ones with pioneer activities (51). The *K*_d_ of LexA and Gal4 on the nucleosomal site are five orders of magnitude higher than that on naked DNA *in vitro* (9). LacO is 20-bp in length, which covers two DNA helical turns and should always be partially occluded on the nucleosome surface regardless of its orientation. Also, given that it is a bacterial TF, LacI is unlikely to have ‘pioneer-factor’ properties that allow it to bind to nucleosome with high-affinity. A potential mechanism for LacI to get over the nucleosome barrier is by taking advantage of the nucleosome dynamics *in vivo*. Events such as DNA replication, histone turnover or nucleosome remodeling can transiently expose DNA, allowing LacI to bind; once it binds, LacI can dwell on the DNA for a long time and prevent the reassembly of nucleosomes (47,52). Consistent with this idea, another bacterial TF, TetR, depletes nucleosome in yeast over its otherwise embedded binding sites (35). Maybe this is the reason why eukaryotic TFs tend to contact less DNA bases and have lower binding energy: nucleosome presents a more formidable barrier for these TFs, so that their binding can be regulated by the chromosome accessibility. Besides nucleosome, other endogenous DNA-binding proteins can also associate with the LacO array non-specifically and interfere with LacI binding. Given the mild effect of nucleosome on LacI binding, we do not expect these highly dynamic and non-specific binding to dramatically increase the apparent *K*_d_ of LacI.

At last, the apparent *K*_d_*in vivo* is likely to be affected by the presence of overwhelming amount of non-specific DNA (53,54). These DNA compete with LacO for LacI:

$$\begin{equation*}{\rm{LacI + LacO}}\mathop{\Longleftrightarrow}^{{K_{d,s}}} {\rm{LacI}} \cdot {\rm{LacO,}}\;{\rm{LacI + gDNA}}\mathop{\Longleftrightarrow}^{{K_{d,n}}} {\rm{LacI}} \cdot {\rm{gDNA}}\end{equation*}$$

Here ‘gDNA’ represents genomic DNA, and *K*_d,s_ and *K*_d,n_ represent dissociation constant on specific and non-specific DNA:

$$\begin{eqnarray*}\frac{{\left[ {{\rm{LacI}}} \right]\left[ {{\rm{LacO}}} \right]}}{{\left[ {{\rm{LacI}} \cdot {\rm{LacO}}} \right]}} = {K_{d,s}},\frac{{\left[ {{\rm{LacI}}} \right]\left[ {{\rm{gDNA}}} \right]}}{{\left[ {{\rm{LacI}} \cdot {\rm{gDNA}}} \right]}} = {K_{d,n}}\end{eqnarray*}$$

It should be noted that the ‘free’ LacI-GFP we measured in this study includes the diffusive part ([LacI]) and the part that is transiently associated with non-specific DNA [LacI·gDNA].

$$\begin{eqnarray*}\left[ {{\rm{LacI}}} \right] + \left[ {{\rm{LacI}} \cdot {\rm{gDNA}}} \right] &=& \left[ {{\rm{LacI}}} \right] + \frac{{\left[ {{\rm{LacI}}} \right]\left[ {{\rm{gDNA}}} \right]}}{{{K_{d,n}}}}\nonumber\\ &=& \left[ {{\rm{LacI}}} \right]^{\prime}\end{eqnarray*}$$

Based on how we defined the apparent *K*_d_, we can convert the *K*_d_ to *K*_d,s_:

$$\begin{eqnarray*}{K_d} &\equiv& \frac{{{{\left[ {{\rm{LacI}}} \right]}^\prime }\left[ {{\rm{LacO}}} \right]}}{{\left[ {{\rm{LacI}} \cdot {\rm{LacO}}} \right]}} \nonumber\\ &=& \frac{{\left[ {{\rm{LacI}}} \right]\left[ {{\rm{LacO}}} \right]}}{{{\rm{[LacI}} \cdot {\rm{LacO]}}}}\left( {1 + \frac{{{\rm{[gDNA]}}}}{{{K_{d,n}}}}} \right) \nonumber\\ &=& {K_{d,s}}\left( {1 + \frac{{\left[ {{\rm{gDNA}}} \right]}}{{{K_{d,n}}}}} \right)\end{eqnarray*}$$

The ratio between *K*_d,s_ and *K*_d,n_ for LacI was shown to be 2.5 × 10^−7^ (53). Depending on how strongly nucleosomes inhibit non-specific LacI binding, 20–100% of the yeast genome can function as competitive DNA (nucleosome coverage of the yeast genome is ∼80%). If we assume this fraction to be 50%, [gDNA] should be about 3.3 mM, which leads to ${K_{d,s}}{\rm{\sim}}{K_d} - 0.8\ {\rm{nM}}$. This calculation shows that the overall apparent *K*_d_ has to be above 0.8 nM due to the presence of non-specific genomic DNA. Our final system with the apparent *K*_d_ <10 nM is approaching this theoretical limit. Interestingly, in *E. coli* cells expressing low-level of LacI dimer (three to five copies per cell), the *t*_on_ and *t*_off_ for an individual Osys were found to be 28 s and 9.3 min, respectively (48). Such binding (∼0.0036 s^−1^nM^−1^) and dissociation rate (0.0018 s^−1^) yield an apparent *K*_d_ of ∼0.5 nM, which is slightly lower than the apparent *K*_d_ we could achieve in yeast (between 0.8 and 10 nM). This is as expected due to more exposed LacO (not chromatinized) and less competitive DNA in the *E. coli* genome.

## Supplementary Material

gkz698_Supplemental_Files
