# Supplementary material for: Enhancement of LacI binding in vivo
Source: Nucleic Acids Res. 2019 Aug 9;47(18):9609–18. doi: 10.1093/nar/gkz698 (PMC6765135; doi:10.1093/nar/gkz698)
Supplement: gkz698_Supplemental_Files [file gkz698_supplemental_files.zip › 1907_Du et al_Supp Figure_NAR2.pdf]

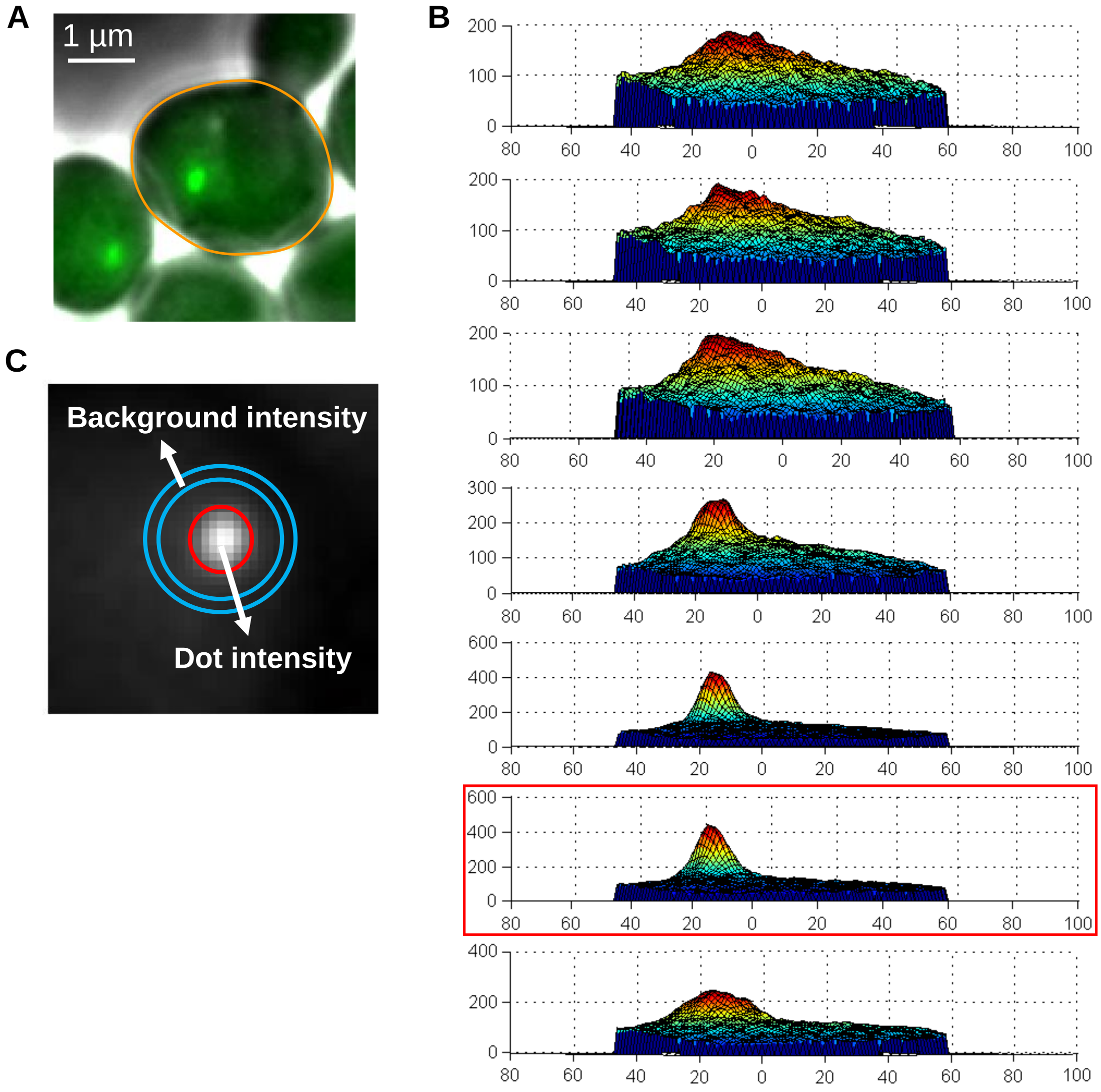

Figure S1. Quantification of dot and background intensity. **A)** Fluorescent images of a cell containing the 256X LacO array and *HIS3* promoter driving GFP-LacI. **B)** Intensity scanning of the fluorescent image in (A) within the cell boundary at different z positions. The red box shows the z position with the sharpest image of the dot (focal position). We deducted the auto-fluorescence intensity from this plot (the whole surface shift down on the z axis for ~20 units), and that is the image we analyzed for the dot and background intensity. **C)** Quantification of dot and background intensity. For each chromatin dot, the background intensity was calculated as the average fluorescence intensity in between the two concentric circles (blue) minus the auto-fluorescence background. The ring is very close to the LacI dot, therefore the GFP intensity in the ring represents the local free protein concentration near the LacO locus. Dot intensity is defined as the volume underneath the peak in B.

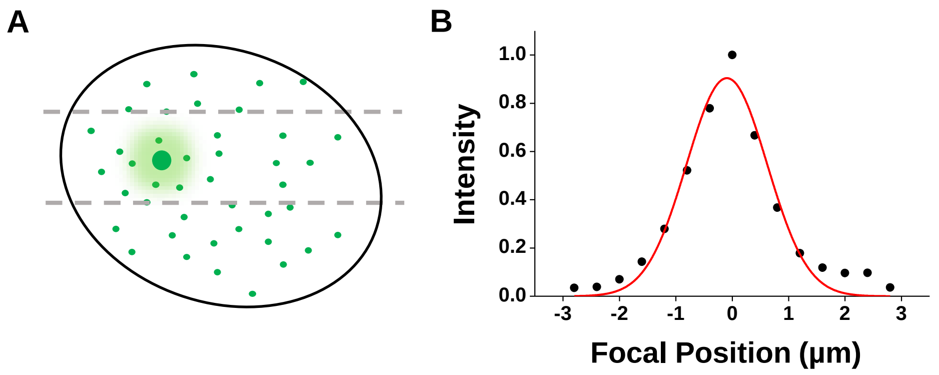

Figure S2. Estimation of Depth of Field (DOF). A) Cartoon illustrating the concept of DOF. Using high NA objective, at each focal point, only a sheet of fluorophores contributes significantly to the fluorescence. **B)** To evaluate the DOF, we calculated the dot intensity as a function of focal positions. At position = 0, the chromosome dot is in focus and therefore generates the largest intensity; the intensity decreases as the focal point moves. The data (black dots) was fitted with a Gaussian, and the area underneath the Gaussian represents the effective DOF (1.6 μm).

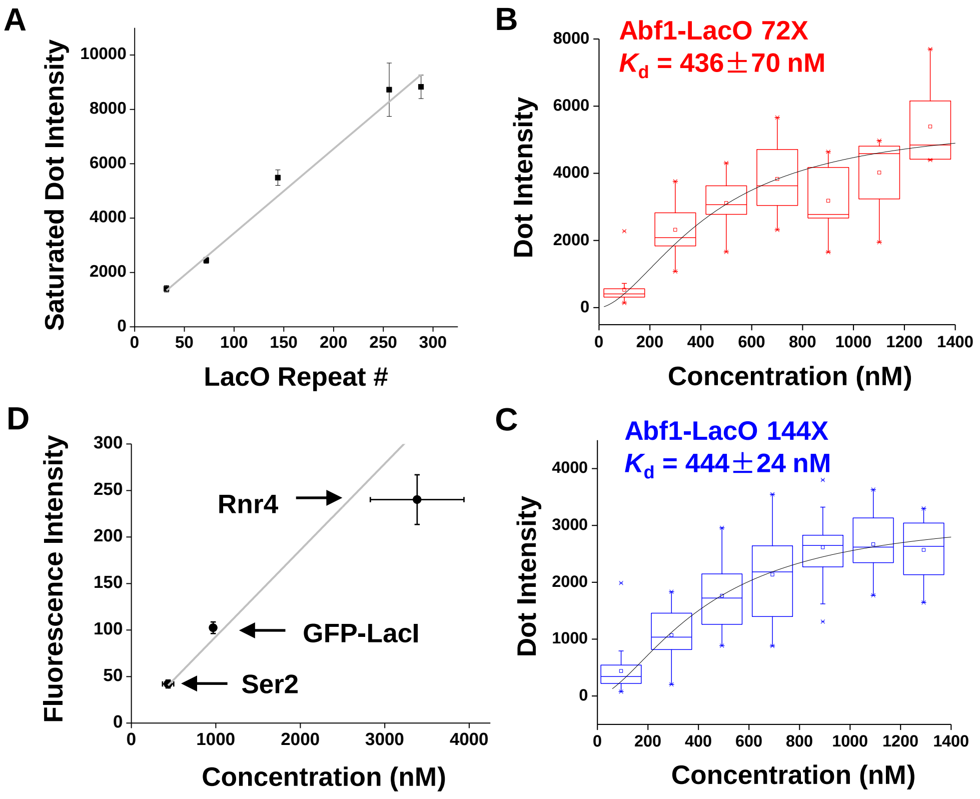

Figure S3. Validation of our measurement of concentration and apparent K_d. A) Saturated dot intensity on five LacO arrays with different repeat number (32, 72, 144, 256, and 288). The intensities scale linearly with the repeat number (Pearson correlation: R = 0.99). **B & C)** Apparent K_d measurement on two of these arrays (72 and 144), and the K_d value is not affected by the array size. *N* = 176 and 117 respectively. **D)** Comparison of our estimated GFP-LacI concentration to previously published concentration of endogenous proteins. We collected eight datasets of protein copy number per cell measured in synthetic media (15-22), and convert them to concentrations using the average haploid yeast volume 42 μm^3 (23). We imaged two GFP-labeled endogenous proteins, Ser2 and Rnr4, and compared their fluorescence intensity and reported concentration to those of GFP-LacI. The fluorescence intensities from the three strains are largely proportional to the concentration of the three proteins. Error bars shown in A & D represent standard error of the mean (SEM), and errors bars in B & C represent 5th to 95th percentile.

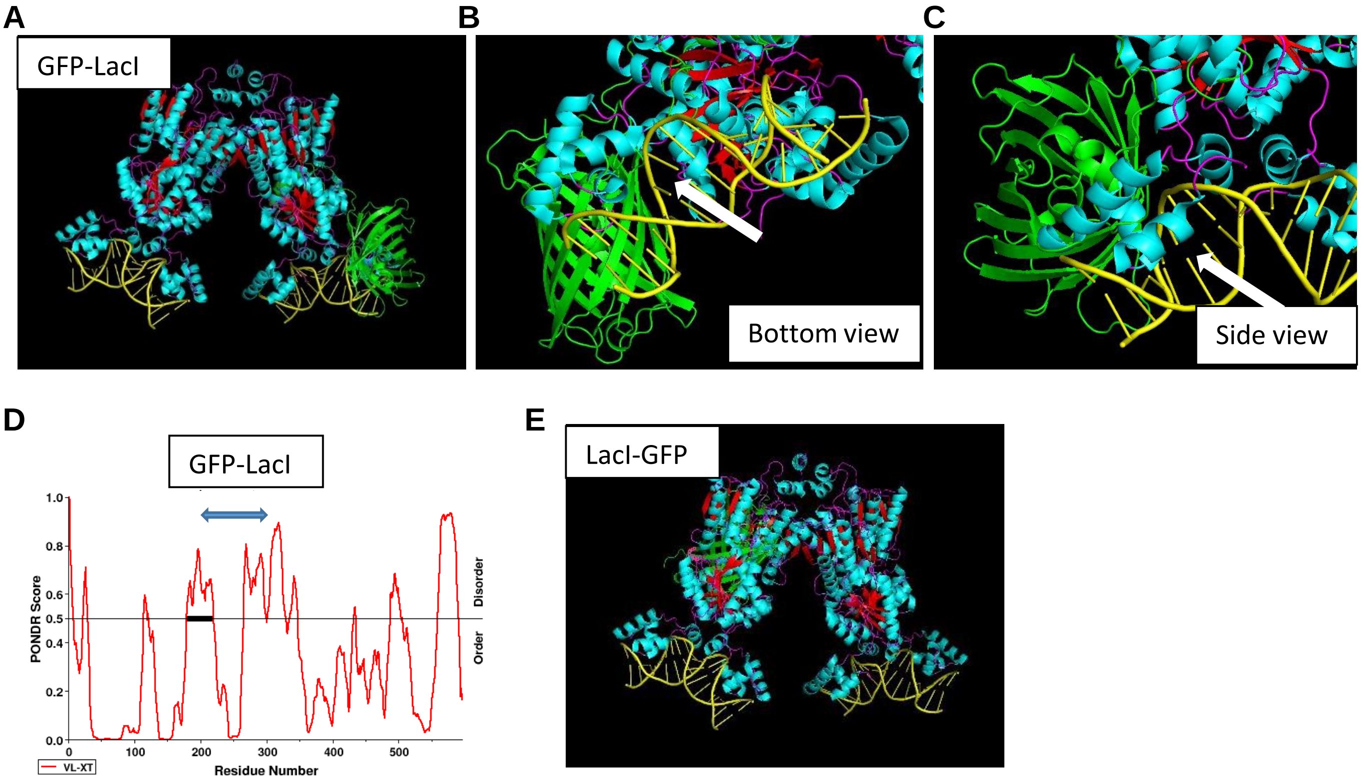

Figure S4. Structural models of GFP-LacI vs LacI-GFP. A-C) Structures of GFP-LacI and DNA. The structure of fusion protein GFP-LacI is modeled through SWISS-MODEL based on the published crystal structures of tetramer LacI and GFP. GFP does not directly block the interaction interface between LacI DNA binding domain and DNA (close views shown in B) and C) where the white arrows point to the LacI-DNA contact). LacI is shown in the cyan-red-purple color. GFP is shown as the green beta barrel. DNA is shown in yellow color. **D)** Predictor Of Naturally Disordered Regions (PONDOR) plots of GFP-LacI. The peaks above the line represent predicted disordered region and the valleys represent structured regions. The linker regions between LacI and GFP indicated by the blue arrows are largely unstructured. **E)** Structure of LacI-GFP and DNA.

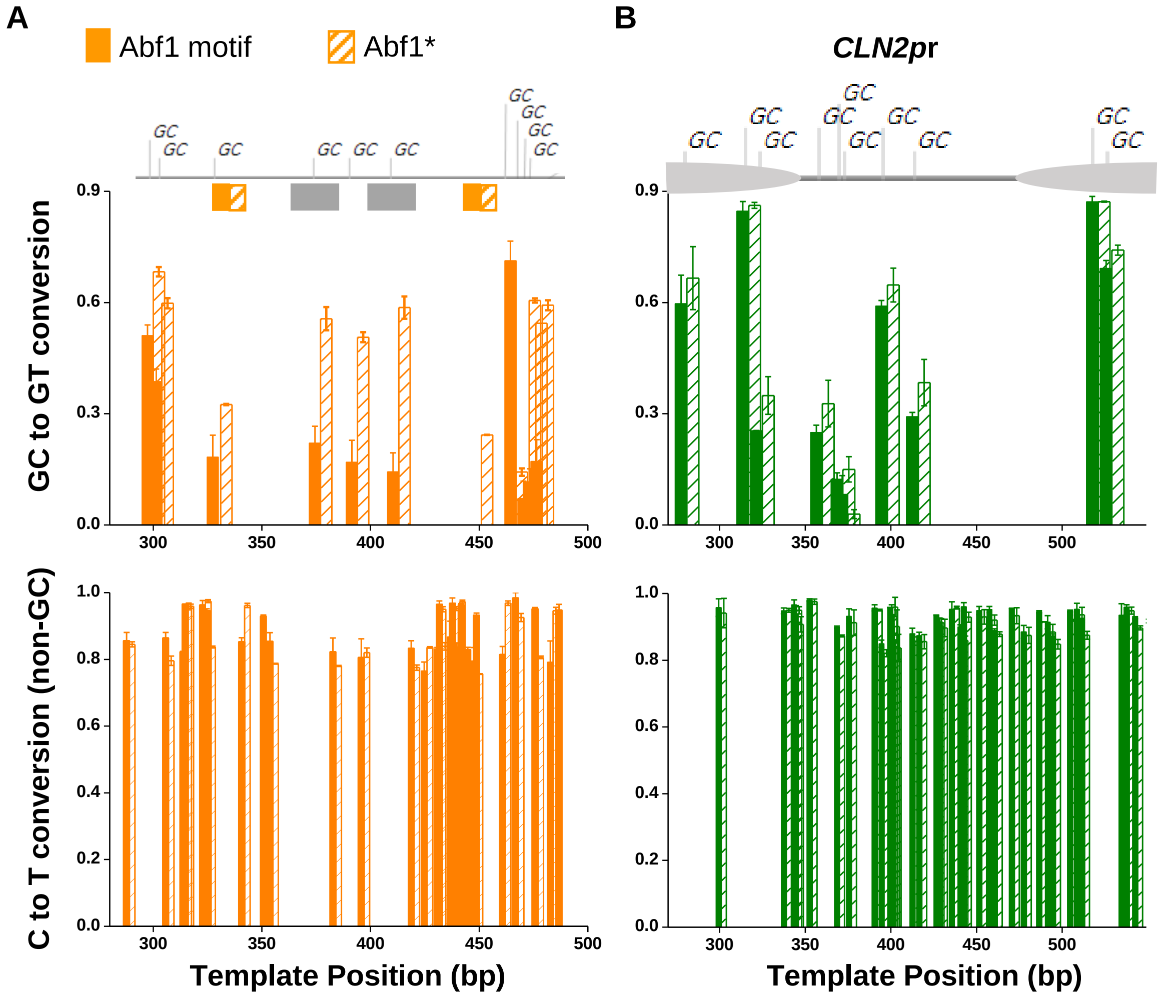

Figure S5. Extra data on the methylation assay. A) The fraction of C converted to T in and out of the "GC" context near the LacO region. The LacO is flanked by either wt (solid) or mutated (hatched) Abf1. The top panel is the same as in Figure 4B, where the GC to GT conversion represents protection from methylation, and the LacO with the Abf1* is more protected than the ones with the wt Abf1. In the bottom panel, the "C"s not in "GC" do not get methylated and should all be converted to "U" during the bisulfite conversion. The high level of "T"s in the sequencing data confirms high efficiency of bisulfite conversion. **B)** The same as in A but over the *CLN2* promoter, a region well-studied for its nucleosome pattern (gray ovals). The data in A and B are from the same cells. In the cells containing LacO with wt (solid) or mutated (hatched) Abf1, the *CLN2* promoter is methylated to a similar extent, indicating that the methyl-transferase was induced to the same level in the two strains. The C to T conversion level in the nucleosomal region (60-85%) is comparable to the level over LacO flanked by Abf1* (~60%); the conversion level in the nucleosome-depleted region (10-30%) is comparable to that over LacO flanked by wt Abf1 (~20%). Error bars here represent SEM.

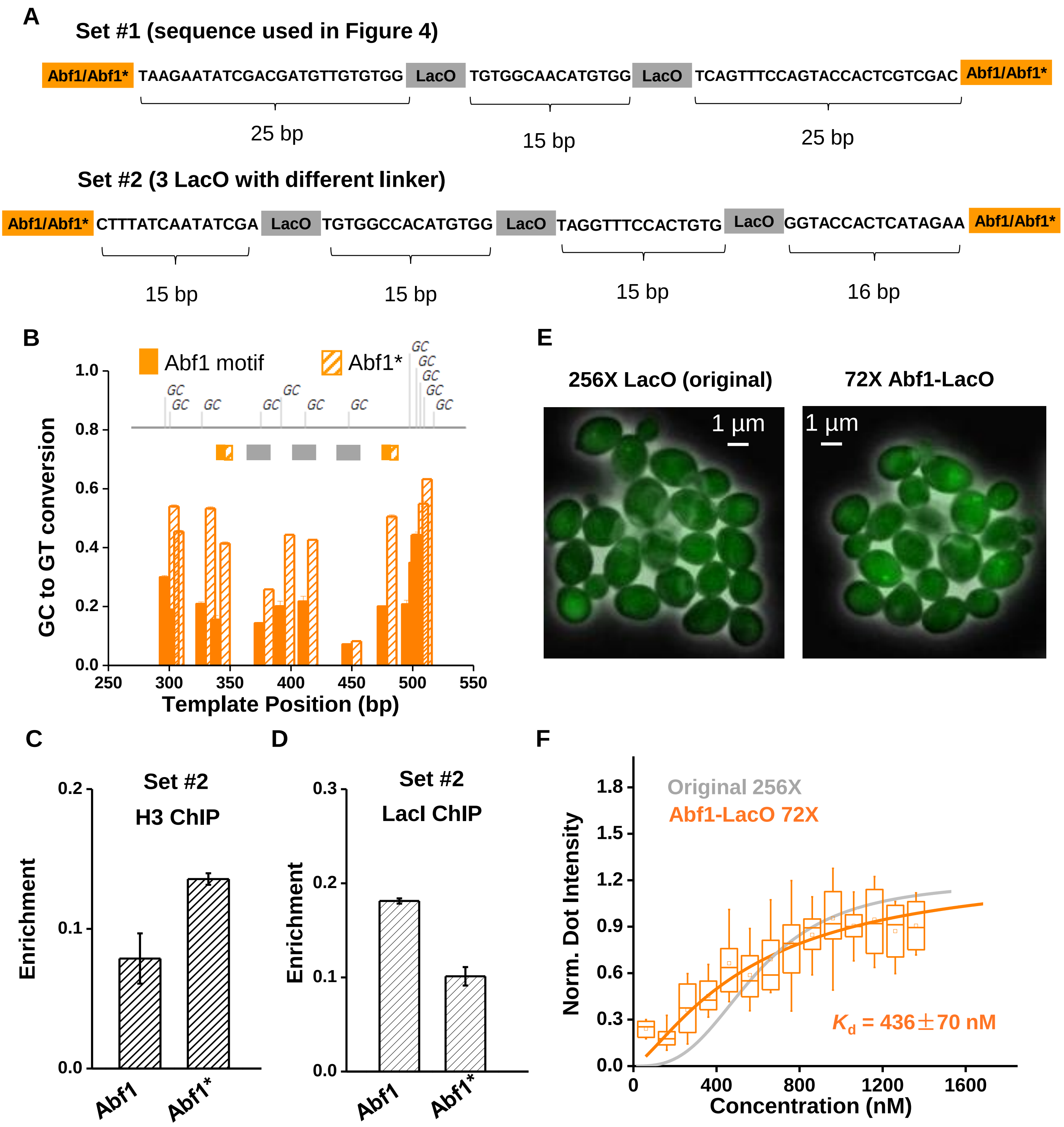

Figure S6. Test of nucleosome effect on LacI binding with templates containing different linker DNA. A) The sequences for two sets of template. **B)** The fraction of "GC" converted to "GT" near the LacO region in the set #2 templates. The LacO is flanked by either wt (solid) or mutated (hatched) Abf1. The LacO with Abf1* is more protected than the ones with the wt Abf1. **C)** H3 enrichment over LacO region in the two set #2 templates. The LacO with Abf1* sites has higher histone enrichment than the LacO with Abf1 sites. **D)** LacI enrichment over the LacO region in the two set #2 templates. The LacO sites with wt Abf1 have higher LacI binding. **E)** Imaging data of *HIS3pr*-GFP-LacI-deg binding on Abf1-LacO array (72X LacO) vs original LacO array (256X). Abf1-LacO array is generated by duplicating the template with the wt Abf1 in set #2 with 24 repeats. We observed brighter dots over the Abf1-LacO array despite the reduced LacO sites. **F)** K_d measurement of the GFP-LacI binding on the two arrays above. The Abf1-LacO array mildly reduces the apparent K_d. 176 cells with the Abf1-LacO array were plotted. Error bars shown in B, C, D represent SEM, and errors bars in F represent 5th to 95th percentile.

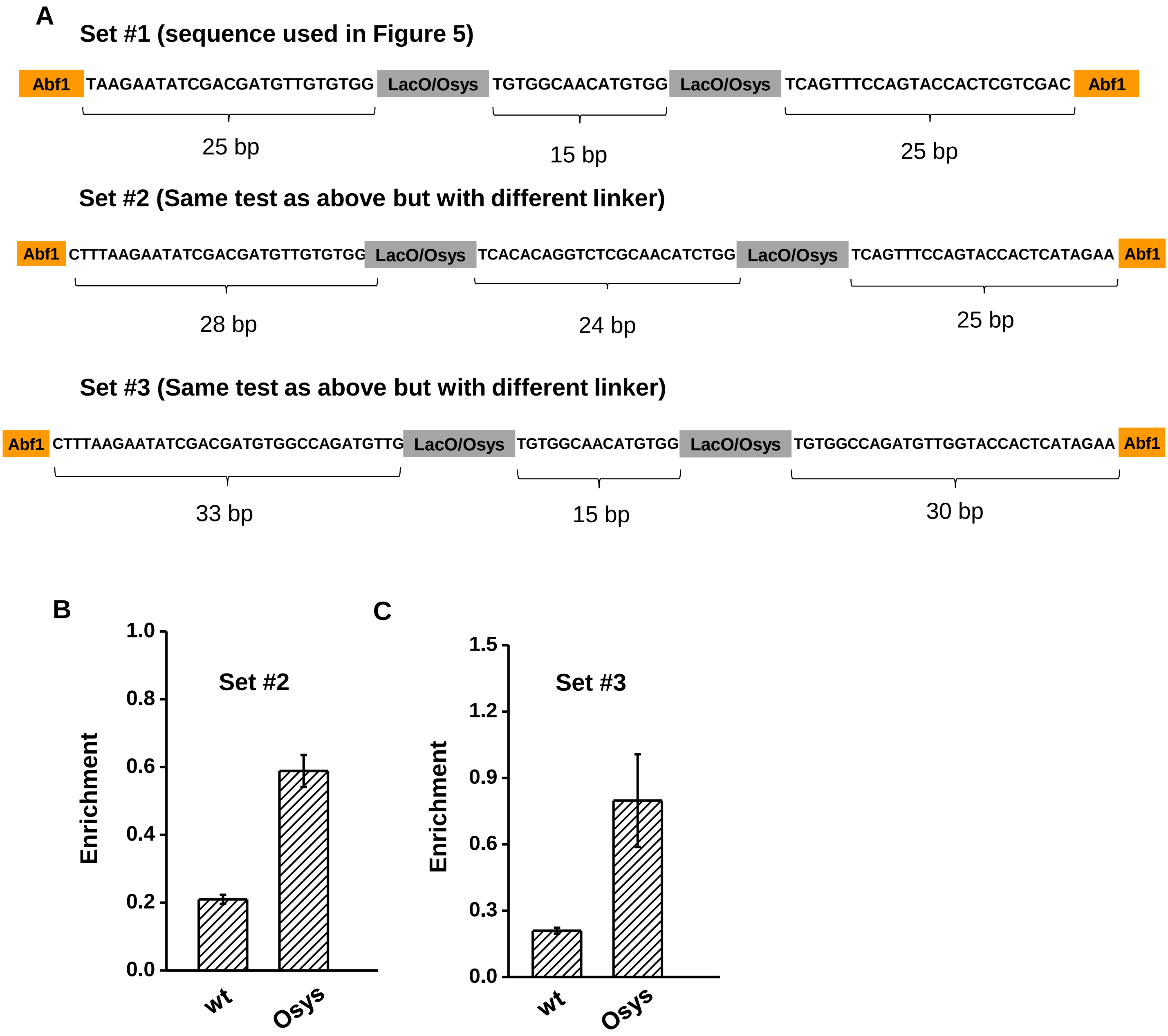

Figure S7. Test the effect of Osys on LacI binding with templates containing different linker DNA. A) The sequence for three sets of templates. Each set has two templates with either wt LacO or Osys. Among the three sets, the Abf1, LacO, Osys elements are kept the same, but the linker sequences are different. **B & C)** LacI enrichment over wt LacO or Osys sites measured by ChIP assay on set #2 & #3 templates. Together with Figure 5C, these data show that Osys leads to enhanced LacI binding regardless of the linker sequence variation. Error bars in B & C represent SEM.

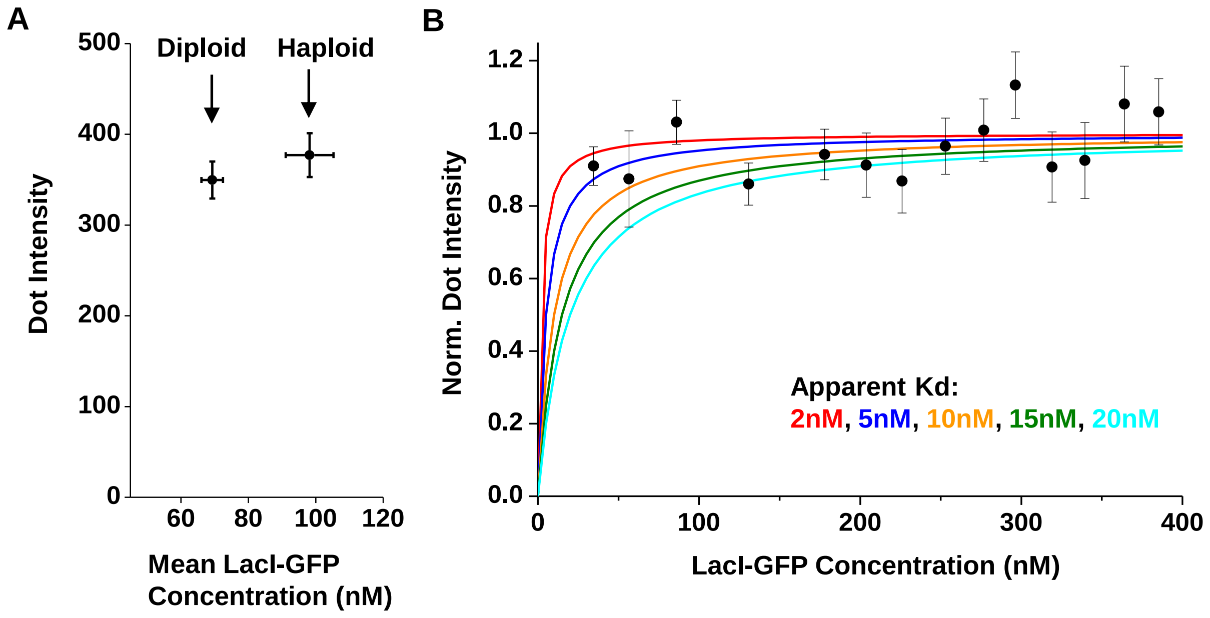

Figure S8. Apparent K_d after combining all genetic traits that favor binding. A) Further reduction of LacI-GFP level by integrating one copy of *REV1pr-LacI-GFP* into diploid cells. In comparison to one copy of *REV1pr-LacI-GFP* into haploid cells, the mean concentration is reduced from ~100 nM to ~70 nM, but the dot intensity is not reduced significantly. **B)** Normalized dot intensity vs LacI-GFP concentration (similar to Figure 6C). The binding curves with apparent K_d of 2, 5, 10, 15, and 20nM are shown for comparison. Error bars represent SEM.
